# Characterizing attitudes related to future child-bearing in young women diagnosed with early-stage breast cancer

**DOI:** 10.1007/s10549-023-07206-5

**Published:** 2024-01-09

**Authors:** Saumya Umashankar, Moming Li, Kaylee Blevins, Mi-Ok Kim, Melanie Majure, John Park, Laura A. Huppert, Michelle Melisko, Hope S. Rugo, Laura Esserman, A. Jo Chien

**Affiliations:** Helen Diller Family Cancer Center, University of California San Francisco, San Franciso, CA USA

**Keywords:** Breast cancer, Young women, Fertility preservation, Endocrine therapy, Family-building in breast cancer

## Abstract

**Purpose:**

This study characterizes attitudes and decision-making around the desire for future children in young women newly diagnosed with early-stage breast cancer and assesses how clinical factors and perceived risk may impact these attitudes.

**Methods:**

This is a prospective study in women < 45 years with newly diagnosed stage 1–3 breast cancer. Patients completed a REDCap survey on fertility and family-building in the setting of hypothetical risk scenarios. Patient, tumor, and treatment characteristics were collected through surveys and medical record.

**Results:**

Of 140 study patients [median age = 41.4 (range 23–45)], 71 (50.7%) were interested in having children. Women interested in future childbearing were younger than those who were not interested (mean = 35.2 [*SD* = 5.2] vs 40.9 years [3.90], respectively, *p* < 0.001), and more likely to be childless (81% vs 31%, *p* < 0.001). 54 women (77.1% of patients interested in future children) underwent/planned to undergo oocyte/embryo cryopreservation before chemotherapy. Interest in future childbearing decreased with increasing hypothetical recurrence risk, however 17% of patients wanted to have children despite a 75–100% hypothetical recurrence risk. 24.3% of patients wanted to conceive < 2 years from diagnosis, and 35% of patients with hormone receptor positive tumors were not willing to complete 5 years of hormone therapy.

**Conclusion:**

Many young women diagnosed with early-stage breast cancer prioritize childbearing. Interest in having a biologic child was not associated with standard prognostic risk factors. Interest decreased with increasing hypothetical recurrence risk, though some patients remained committed to future childbearing despite near certain hypothetical risk. Individual risk assessment should be included in family-planning discussions throughout the continuum of care as it can influence decision-making.

## Introduction

Young women diagnosed with invasive breast cancer at less than 45 years of age represent about 10.3% of all women diagnosed breast cancers in the United States [1]. Premenopausal women face unique challenges, including the impact of oncologic treatments on fertility and future family-planning.

Breast cancer treatment can have direct and indirect effects on fertility. Chemotherapy causes gonadotoxicity, and patients can be relatively less fertile following treatment despite the resumption of regular menses [2–5]. For young women with hormone receptor-positive (HR +) breast cancer, a minimum of 5 years of adjuvant endocrine therapy is the standard of care and both reduces the risk of recurrence and improves survival [6]. While endocrine therapy (ET) is not known to cause direct gonadotoxicity [7], pregnancy is contraindicated for patients on ET due to the risk of teratogenic effects [8]. This contraindication can result in some patients choosing to delay pregnancy, putting patients at risk for age-related infertility [9]. Studies have shown that fertility concerns impact patient decision-making and adherence to adjuvant endocrine therapy [10, 11].

Partridge et al. confirmed that fertility after treatment is a major concern for young women with breast cancer [12]. In a web-based survey, researchers found that nearly one-third of patients reported that fertility concerns and a desire to have more children in the future affected their treatment decisions. Although referral to a fertility specialist is now part of the American Society for Clinical Oncology (ASCO) and National Comprehensive Cancer Network (NCCN) guidelines for young women, a number of studies have reported inadequate reproductive counseling [13–15]. These results reinforce the need for care teams to better understand the spectrum of patient attitudes and preferences related to future biologic children and how clinical factors impact patient decision-making.

The impact of understanding individual risk of breast cancer recurrence on desire for future biologic children and treatment decision-making is not well characterized [10, 12]. We conducted a prospective survey study in young women with early-stage breast cancer to assess these attitudes as well as their impact on preferred timing of future pregnancy.

## Methods

### Patients, recruitment, and data collection

In February 2018, the University of California, San Francisco (UCSF)’s Breast Care Center (BCC) established a registry of young women diagnosed with invasive breast cancer at less than 45 years of age. Young women were prospectively identified through the breast medical oncology clinic schedules. Women with early-stage breast cancer (stage I–III) who were within 6 months of diagnosis and were English-speaking were eligible to participate in this prospective survey study assessing the attitudes and decision-making of young women with early-stage breast cancer.

Eligible patients were contacted by email and/or phone. Interested patients were emailed a consent form using DocuSign. Those who signed consent were emailed a survey. Surveys were created, managed, and distributed electronically via REDCap® to patients. Patients received up to four email reminders to complete the survey, including one direct patient contact by a research coordinator.

The baseline survey assessed attitudes towards decision-making across a variety of topics considered to be relevant to young women with breast cancer including fertility and family-building, career, relationships, financial toxicity. Anxiety, depression, fear of recurrence, quality of life, cognitive function, and sleep were assessed through validated PROMIS measures. Patient demographics, clinicopathologic data, and treatment history were obtained and confirmed through the electronic medical record. Surveys were distributed at baseline and annually. This study reports the results from the fertility and family building sections of the baseline survey.

For patients that were interested in future childbearing, attitudes around future childbearing and family building were assessed using hypothetical risk scenarios. First, we asked how hypothetical risk of recurrence of their breast cancer (which may not be curable at that time), assessed through hypothetical absolute risk of recurrence brackets (0%, 1–4%, 5–9%, 10–24%, 25–49%, 50–74% and 75–100%) impacted their desire to want to have biological children (assessed through a binary response) from the patients. Second, we asked, if, hypothetically, pregnancy itself increased patients’ baseline risk of recurrence by the hypothetical percentage brackets (0%, 1–4%, 5–9%, 10–24%, 25–49%, 50–74% and 75–100%), if they would choose to get pregnant, use a surrogate or not have biological children. Lastly, in patients with HR+ tumors, we asked if early discontinuation of endocrine therapy increased patients’ baseline risk of recurrence by the hypothetical percentage brackets (0%, 1–4%, 5–9%, 10–24%, 25–49%, 50–74% and 75–100%), if patients would still seek to discontinue endocrine therapy early to try to conceive themselves, complete 5 years of endocrine therapy while conceiving via a surrogate during those 5 years, or complete 5 years of endocrine therapy and try conceiving themselves after completing of endocrine therapy.

Approval for the study was obtained from the ethics committee at the University of California, San Francisco. Informed consent for study participation and sharing of de-identified data were obtained from all individuals participating in the study.

### Statistical analyses

Descriptive statistics were used to summarize demographic and clinical data for young women that participated in the survey study. Independent-samples *t*-test and chi-square tests were conducted to compare demographics and clinical characteristics for young women who were interested in having children in the future vs those that were not interested in having children in the future.

Patient’s attitudes towards family building and fertility preservation were summarized using descriptive statistics. Ordinal logistic regression was used to assess the effects of patient, disease, and treatment factors independently one by one on patients’ acceptance of the highest level of recurrent risk for their own or surrogacy pregnancy. With no evidence of violation of the proportional odds assumption, the effects of each factor on the odds of accepting a higher recurrence risk level, compared to a lower or equal risk, were assumed constant. For the non-negligible missing responses (13%), we performed both complete case only and missing data analysis using the inverse of probability weighting (IPW) method [16]. Assuming that the missing responses were randomly missing after accounting for differences in the patient/disease characteristics (i.e. missing at random) the missing data analysis yielded similar results as the complete case only analyses.

All statistical analyses were performed using R and RStudio, version 3.6.3 and 1.2.5033 respectively.

## Results

### Patient population

**Fig. 1:**
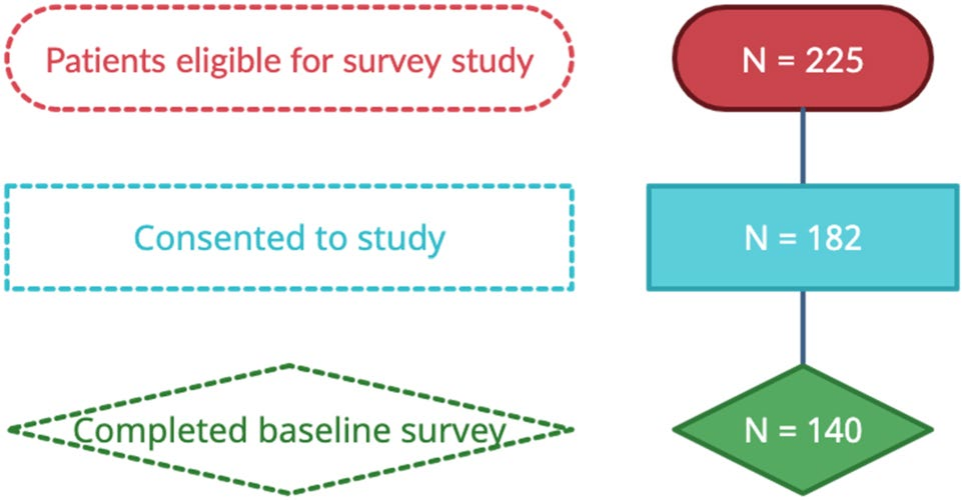
Consort diagram

Between February 2018 and February 2022, 225 patients with early-stage breast cancer diagnosed within 6 months of study entry were identified through the UCSF breast medical oncology clinic schedules and were eligible to participate in this prospective survey study. Of these patients, 182 patients consented to participate in the study and 140 patients completed the baseline survey (completion rate = 77%, Fig. 1). Demographic and clinical data for the patients that completed the baseline survey are summarized in Table 1. The mean age of patients who completed the survey was 41.4 years (range 23–45). The median age for the 42 patients that did not complete the survey was 39.9, range 29–44). There was no significant difference in the age, tumor receptor status, and treatment (chemotherapy vs. no chemotherapy, hormone therapy use) between the patients that completed the survey and those that did not complete the survey. 12% of patients were BRCA1/2 positive and 7% had some other pathogenic mutation(s). The majority of patients who were enrolled to the study and completed the surveys were white (62.1%), educated (83.6% college graduates or above), employed (69.3% full time, 10.0% part-time), and 62.9% had an annual income of > $100,000 as self-reported via surveys. 80.8% of participants were partnered (married, domestic partnership, or committed relationship).

**Table 1:** Patient and tumor characteristics

	Overall (*N* = 140)	Interested in children in the future (*N* = 71)	Not interested in children in the future (*N* = 69)	*p* value
Age (years)				
Mean (SD)	38.0 (5.36)	35.2 (5.12)	40.9 (3.90)	<0.001
Median [min, max]	39.1 [23.4, 45.0]	35.8 [23.4, 44.9]	41.4 [24.2, 45.0]	
Stage^a^				
1	33 (24.1%)	14 (20.0%)	19 (28.4%)	0.133
2	70 (51.1%)	33 (47.1%)	37 (55.2%)	
3	33 (24.1%)	22 (31.4%)	11 (16.4%)	
Hormone receptor status				
Negative	45 (32.1%)	28 (39.4%)	17 (24.6%)	0.0904
Positive	95 (67.9%)	43 (60.6%)	52 (75.4%)	
HER2 status				
Negative	102 (74.5%)	55 (78.6%)	47 (70.1%)	0.35
Positive	35 (25.5%)	15 (21.4%)	20 (29.9%)	
Grade				
1	17 (12.7%)	9 (13.0%)	8 (12.3%)	0.107
2	54 (40.3%)	22 (31.9%)	32 (49.2%)	
3	63 (47.0%)	38 (55.1%)	25 (38.5%)	
Nodal involvement^b^				
Negative	74 (54.0%)	39 (55.7%)	35 (52.2%)	0.813
Positive	63 (46.0%)	31 (44.3%)	32 (47.8%)	
Chemotherapy				
No	26 (19.5%)	11 (15.7%)	15 (23.8%)	0.339
Yes	107 (80.5%)	59 (84.3%)	48 (76.2%)	
Hormone therapy				
No	41 (33.1%)	25 (36.8%)	16 (28.6%)	0.439
Yes	83 (66.9%)	43 (63.2%)	40 (71.4%)	
Genetic mutations				
Negative	107 (81.1%)	54 (78.2%)	53 (84.1%)	0.588
BRCA1/2 positive	16 (12.1%)	10 (14.5%)	6 (9.52%)	
Other pathogenic mutation^c^	9 (6.8%)	5 (7.2%)	4 (6.3%)	
Relationship status				
Married or domestic partnership	88 (62.9%)	33 (46.5%)	55 (79.7%)	0.033^**^
Committed relationship	25 (17.9%)	19 (26.8%)	6 (8.7%)	
Single	17 (12.1%)	14 (19.7%)	3 (4.3%)	
Divorced or separated	9 (6.4%)	4 (5.6%)	5 (7.2%)	
Other	1 (0.7%)	1 (1.4%)	0 (0%)	
Prior children				
No	62 (44.3%)	49 (69.0%)	13 (18.8%)	<0.001
Yes	78 (55.7%)	22 (31.0%)	56 (81.2%)	
Education				
High school or less	6 (4.3%)	3 (4.2%)	3 (4.3%)	0.108
Some college or technical school	15 (10.7%)	4 (5.6%)	11 (15.9%)	
College degree	56 (40.0%)	34 (47.9%)	22 (31.9%)	
Post-graduate degree	61 (43.6%)	30 (42.3%)	31 (44.9%)	
Income				
$0–$25,000	4 (2.9%)	3 (4.2%)	1 (1.4%)	0.232
>$25,000–$50,000	11 (7.9%)	8 (11.3%)	3 (4.3%)	
>$50,000–$75,000	9 (6.4%)	3 (4.2%)	6 (8.7%)	
>$75,000–$100,000	22 (15.7%)	10 (14.1%)	12 (17.4%)	
>$100,000–$200,000	40 (28.6%)	23 (32.4%)	17 (24.6%)	
>$200,000	48 (34.3%)	23 (32.4%)	25 (36.2%)	
Employment				
Full-time employment	97 (69.3%)	56 (78.9%)	41 (59.4%)	0.151
Part-time employment	14 (10.0%)	6 (8.5%)	8 (11.6%)	
Full-time stay-at-home parent	12 (8.6%)	2 (2.8%)	10 (14.5%)	
On disability or leave of absence	3 (2.1%)	1 (1.4%)	2 (2.9%)	
Student	2 (1.4%)	1 (1.4%)	1 (1.4%)	
Unemployed	7 (5.0%)	3 (4.2%)	4 (5.8%)	
Other	3 (2.1%)	2 (2.8%)	1 (1.4%)	
Race				
White	87 (62.1%)	45 (63.4%)	42 (60.9%)	0.8
Asian	34 (24.3%)	18 (25.4%)	16 (23.2%)	
Black or African American	4 (2.9%)	3 (4.2%)	1 (1.4%)	
Other	21 (14.9%)	10 (14.1%)	11 (15.8%)	

**p value was calculated by merging categories into partnered (married, domestic partnership, or committed relationship) vs. unpartnered (single, divorced, separated)

^a^Defined as clinical stage for neoadjuvant patients and pathological stage for patients who did not receive neoadjuvant therapy

^b^Defined as clinically node positive for neoadjuvant patients, pathologically node positive for patients not receiving neoadjuvant therapy

^c^Including CHEK2, ATM

Of the 140 patients that completed the baseline survey, 71 patients (50.7%) were interested in having a child in the future. Comparisons of demographics and clinical characteristics for young women who were interested in having children in the future vs those that were not interested in having children in the future are summarized in Table 1. Patient age and whether the patient already had children were the biggest drivers of whether young women were interested in future biologic children vs. not. Patients who were interested in future children were younger than those who were not interested in having future children [mean = 35.2 years (*SD* = 5.12) vs 40.9 years (*SD* = 3.90), *p* < 0.001], and more likely to be childless compared to those who were not interested in future children (81.2% vs 31.0%, *p* < 0.001). Of the 67 women that already had a child, 21 (31.3%) were interested in future children. Women who were interested in child bearings were more likely to be single compared to women who were not interested in future children (25.3% vs 11.5%, *p* = 0.033), though this association may be confounded by younger age. Clinicopathologic factors such as disease stage, treatment (chemo vs no chemo), tumor grade, nodal status, receptor status and presence of hereditary genetic mutations were not associated with interest in having future children.

Of the 69 women who were not interested in future children 51 (74%) felt that their family was complete, 9 (13%) did not desire having children, 4 (5.8%) feared that having children would negatively impact their breast cancer outcome, 3 (4.3%) had a genetic mutation that they did not want to pass to their children, and 2 (2.9%) replied other.

### Attitudes towards family-building

**Fig. 2:**
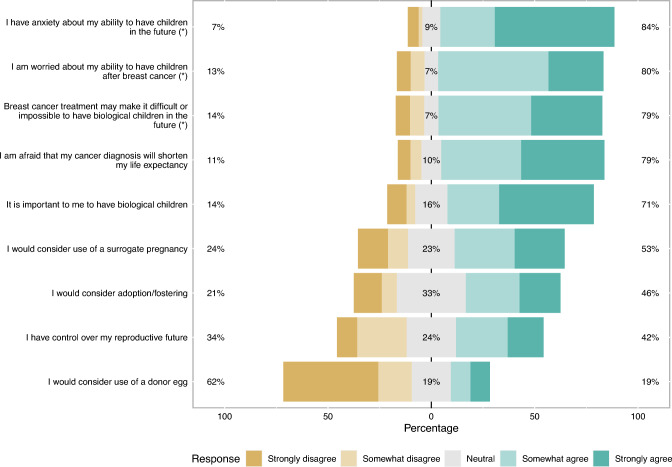
Family-building attitudes in young women. Asterisk: questions that were asked only for patients that were interested in having future children

The 140 patients who completed the baseline survey were asked additional questions regarding attitudes around a variety of topics relating to family-building options, cancer-related anxiety and its impact on family building using Likert Scale responses; results are summarized in Fig. 2. Of the patients that completed the survey, 79% agreed that they were concerned about their cancer diagnosis shortening their life expectancy. Patients who were concerned about a shortened life expectancy were less likely to indicate interest in having children in the future (57.9% vs 71.4%, *p* = 0.02). The majority of patients would consider use of a surrogate pregnancy (53%) and adoption/fostering (46%), however only a minority (19%) would consider use of a donor egg. Seventy nine percent of the 71 patients that were interested in having children in the future were concerned that treatment would make it difficult for them to have biological children, and 84% of these patients reported that they have anxiety about their ability to have children in the future. 54 patients (38.6% of overall population and 77.1% of patients interested in child-bearing) had already undergone or planned to undergo fertility preservation and cryopreservation.

**Fig. 3:**
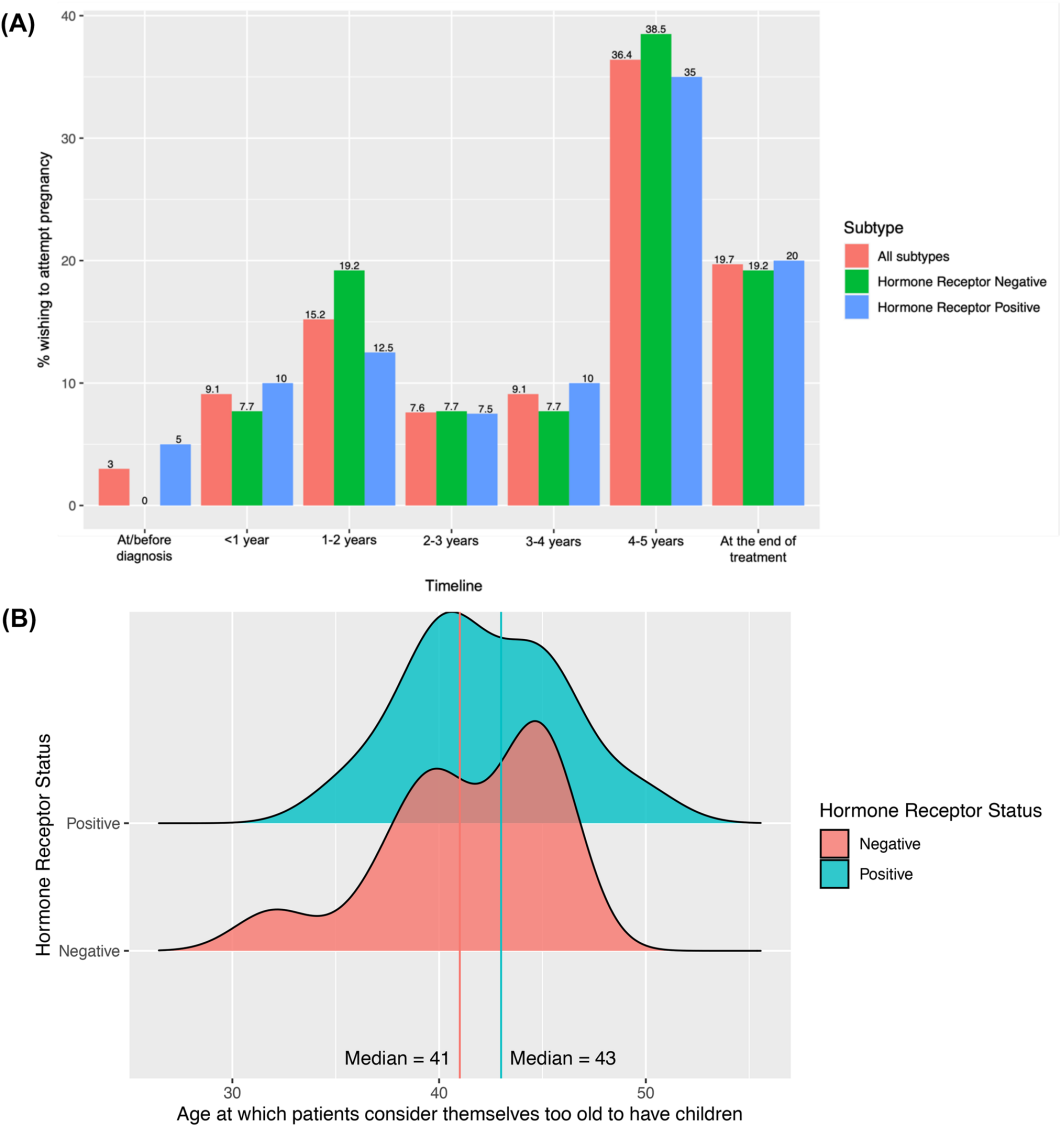
**A** Ideal timing for attempting pregnancy in relationship to breast cancer diagnosis. **B** Age at which patients consider themselves too old to have children

The 71 women interested in having a biologic child in the future were asked about preferences in timing of future pregnancy. 19.7% were willing to wait until the recommended adjuvant treatment was complete, even if more than 5 years. 36.4% were willing to wait 4–5 years. 24.3% were interested in trying to conceive within the first 2 years of diagnosis. There was no significant difference in timing preferences by tumor receptor subtype (Fig. 3). Women were asked about the age at which they perceive themselves to be too old to have a biologic child. The median age was 43 for patients with hormone receptor positive (HR+) breast cancer (median age at diagnosis = 35.9), and 41 for hormone receptor negative (HR−) breast cancer (median age at diagnosis = 35.7).

### Hypothetical risk of recurrence and desire for future children

**Fig. 4:**
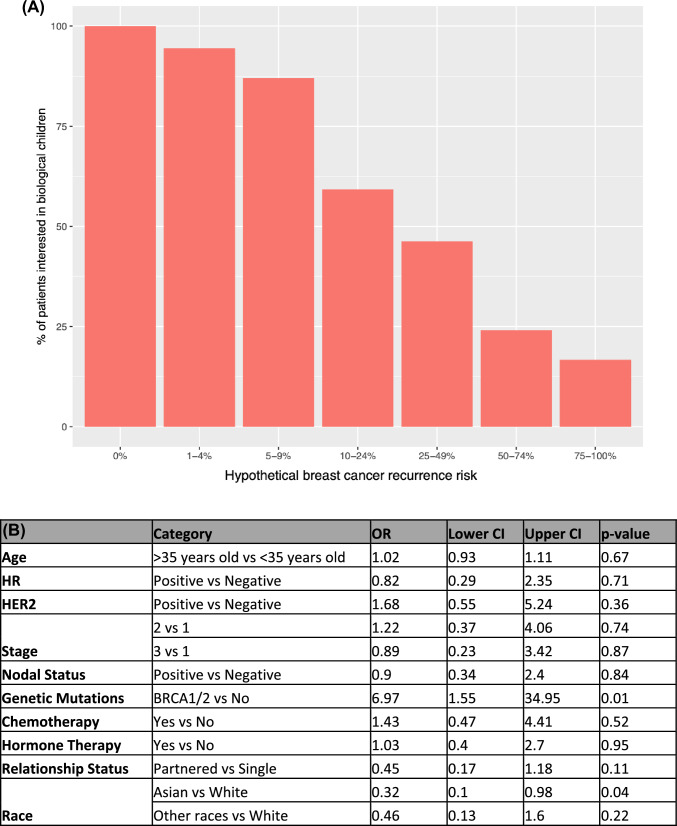
**A** Interest in future biologic children with different hypothetical scenarios of breast cancer recurrence risk. **B** Odds ratios associated with patients that were more vs less risk averse based on patient characteristics

**Fig. 5:**
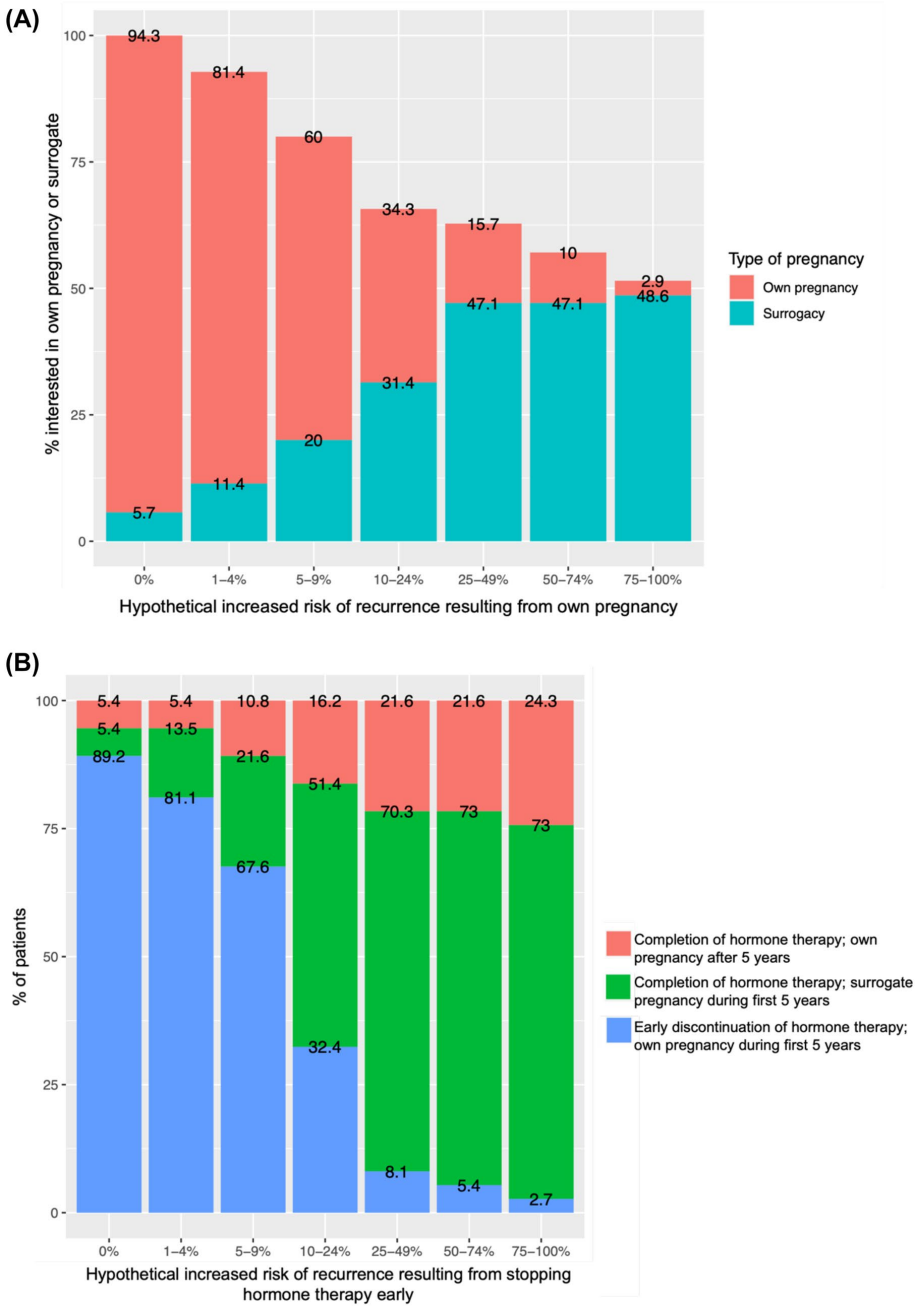
**A** Interest in own or surrogate pregnancy based on different hypothetical scenarios of increased risk resulting from pregnancy. **B** Interest in early discontinuation vs completion of hormone therapy based on different hypothetical scenarios of increased risk of recurrence resulting from early discontinuation of hormone therapy

Patients interested in childbearing were presented with hypothetical scenarios of recurrence risk and again asked about their interest in future biologic children. Results are summarized in Fig. 5. Patient’s desire for future children decreased with increasing hypothetical risk, with the greatest shift from having interest to not having interest when there was a 10–24% risk of recurrence. Of note, 16.4% of young women were interested in future children even when presented with a 75–100% hypothetical risk of recurrence, and nearly a quarter of patients were interested in future biologic children when presented with a hypothetical 50% or greater risk of recurrence. With increasing risk of recurrence, Asian women were less interested in future children compared to white women (OR 0.32 vs white young women, *p* = 0.04) while BRCA-carriers were more likely to remain interested in future children with increasing risk (OR 6.97  vs no genetic mutation, *p* = 0.01) (Fig. 4). There was no association between other clinicopathologic factors or demographics and interest in future children when presented with hypothetical risk scenarios (Fig. 4B).

### Hypothetical risk of pregnancy on recurrence and desire for own pregnancy vs surrogate pregnancy

Patients were reminded that there are currently no data showing that pregnancy is associated with increased risk of recurrence or worse prognosis in patients with early-stage breast cancer, however these data (at the time of this study) were limited to retrospective cohort studies and single arm studies [17]. The 71 patients who were interested in future children were presented with hypothetical scenarios of risk associated with pregnancy and asked about their continued interest in getting pregnant vs interest in using a surrogate to carry the pregnancy. With increasing hypothetical risk of recurrence due to pregnancy, patients were less likely to want to carry their own pregnancy, and more likely to be interested in surrogacy or not getting pregnant (Fig. 5A). The majority of patients continued to be interested in carrying a pregnancy when the hypothetical increased absolute risk of recurrence due to pregnancy was < 10%, however most shifted preference when presented with a hypothetical increased absolute risk of 10% or greater.

When looking specifically at patients with HR + disease, 43 of 71 patients who were interested in child-bearing had HR + breast cancer. Of these 43 patients, 40.8% of patients responded that they would be willing to complete 5 years of hormone therapy before trying to conceive, 24.5% would consider completing 5 years of hormone therapy before trying to conceive, and 34.7% would not be willing to complete 5 years of hormone therapy before trying to conceive. These 43 women were then given hypothetical scenarios of risk of recurrence related to stopping hormone therapy early and asked about their interest in stopping hormone therapy early to try to conceive vs. choosing to have a surrogate pregnancy or no pregnancy. Patients were less likely to discontinue hormone therapy early to have their own pregnancy as the hypothetical risk of recurrence due to early discontinuation of hormone therapy increased. The majority of patients (67.6%) would discontinue hormone therapy early to try to conceive if early discontinuation resulted in < 10% increased absolute risk of recurrence. However, if early discontinuation hypothetically increased risk of recurrence by 10% or greater, the majority of patients would elect to have a surrogate pregnancy and complete 5 years of hormone therapy in order to have a child within the first 5 years of diagnosis (Fig. 5B).

## Discussion

In this analysis of prospectively collected survey data from a single large academic medical center of 140 young women diagnosed with early-stage breast cancer before age 45, the interest in future biologic children was high, with over half the population indicating a desire to have a biologic child in the future. The desire for children in the future was associated with younger age and being child-less, and was independent of clinical factors such as disease stage, nodal status, receptor status, tumor grade, and treatment (i.e., chemotherapy receipt vs. not) suggesting that traditional prognostic factors associated with risk of recurrence did not seem to influence the desire for future children. Our findings are consistent with other studies showing that women who had children before cancer are less likely to undergo fertility preservation, and nulliparity is a significant predictor of concern about future fertility [13, 14].

In our study, 77% of patients who were interested in future children underwent embryo/oocyte preservation. The use of fertility preservation techniques in our population was significantly higher compared with previous early reports of fertility preservation in young women, but comparable to more recent cohorts [15, 18, 19]. Fertility preservation has not been shown to impact breast cancer prognosis and treatment outcomes, and can be completed without significant delays in treatment initiation [11, 20]. Nonetheless, 79% remained concerned about their ability to have biological children after treatment, and 84% reported anxiety about their ability to have children in the future. The elevated anxiety and concern around ability to have future biological children despite the high use of embryo/oocyte preservation techniques suggests a persistent unmet psychological need in this population despite taking action to preserve fertility.

Our data provide increased understanding of patients’ desire to have future children in the context of one’s understanding of their individual risk. Of the patients that were not interested in future children, only a minority (< 10%) did not want more children due to reasons related to their breast cancer. Our survey results show that interest in future child-bearing decreases when patients are presented with increasing hypothetical risk of recurrence. Interestingly, the trend of decreasing interest in future children with increasing risk of recurrence was not impacted by age, receptor status, stage, or type of systemic therapy suggesting that standard prognostic clinicopathological factors did not impact individual desire for future children. These findings suggest that traditional validated prognostic risk factors are not good predictors for a patient’s desire to have a future child, however having a clear quantitative assessment of risk of recurrence does appear to influence a patient’s desire and decisions around family-building. This is interesting because it implies that patients may not be aware or fully understand the clinical relevance of specific clinicopathologic factors that providers use to assess risk of recurrence, and suggests that a more direct concrete understanding of their individual risk in the context of family-building discussions may be helpful.

This study highlights the importance of timing of pregnancy for patients. The median age at which patients felt they were too old to have a child is 42 years, which is only 4 years older than the median age at study entry. This may be driving a sense of urgency. At baseline, almost half the patients interested in biological children would like to start trying to conceive within 2–3 years. Only 20% of patients wished to wait 5 years prior to trying to conceive, and a quarter of patients were interested in trying to conceive in the first 1–2 years. This is most clinically relevant in patients with HR + tumors where the current standard of care is to take a minimum of 5 years of adjuvant endocrine therapy during which pregnancy is contraindicated. In this study, of the 43 patients with HR + tumors, 35% were not willing to complete the full 5 years of hormone therapy prior to trying to conceive.

The risks, if any, associated with pregnancy and interruption or early discontinuation of hormone therapy fall into the category of questions that will always be difficult to answer definitively in a prospective randomized trial. This study suggests that an attempt to estimate what those risks might be may impact both medical and reproductive decision making. Our results show that patients are less interested in interrupting or discontinuing hormone therapy early if it would increase risk of recurrence, and the majority of patients would reconsider if the increased absolute risk from treatment interruption surpasses 10%. Our data also show that, in these circumstances, more patients would rather pursue a surrogate pregnancy within the first 5 years rather than wait 5 years to complete hormone therapy in order to carry the pregnancy themselves.

These data are timely considering the recent results from the POSITIVE trial. The POSITIVE trial is a single-arm phase II prospective study of premenopausal women with early-stage HR + breast cancer who had received 18–30 months of adjuvant hormone therapy and wished to interrupt hormone therapy to attempt pregnancy [21]. Patients were allowed to interrupt hormone therapy for a maximum of 2 years including conception, pregnancy, delivery, and breastfeeding. In the 516 patients enrolled with a median of 41 months follow up, the 3-year incidence of breast cancer events was below the pre-specified safety threshold at 8.9%, and was not statistically different from the recurrence rate seen in the external control population from the SOFT/TEXT trial [22, 23]. 368 patients (74%) had at least 1 pregnancy with 317 (63.8%) having at least one live birth. This study is the first to provide prospective data regarding the potential safety of adjuvant hormone therapy interruption during the first 5 years after diagnosis. These data are encouraging, however long-term follow up is needed for this population since these patients are at risk of later recurrence. Our data support the importance of including individual risk assessment when having discussions with patients about pregnancy, timing of pregnancy, and potential interruption of systemic therapy.

This study had several strengths. This study incorporated hypothetical risk scenarios to demonstrate how discussion of risk in concrete terms can have strong influence on one’s medical and reproductive decision-making. This study had a high survey response rate that was complemented by detailed chart review to provide a complete picture of both patient preference and clinical characteristics. This is a prospective study that plans to continue to follow patients longitudinally with annual surveys in order to assess how their desire and plans for future children change over time.

Our study has several key limitations. First, the study was conducted at a single institution and major academic medical center. Most patients were white, highly educated, and of high socioeconomic status. These patients likely have greater resources which enables increased options for fertility preservation, support during and after pregnancy, and the ability to explore surrogacy options which can be expensive and complex. The rates of fertility preservation and interest in the potential use of a surrogate likely reflects this unique demographic which potentially makes our results less generalizable to other populations. Future studies can expand this work in a more diverse population with regard to race/ethnicity, socioeconomic status, and other demographic features.

Desire to have a child after a diagnosis of early breast cancer is strong and should be acknowledged when discussing treatment options and goals throughout the continuum of care. It is not only important to have upfront conversations around fertility preservation, but also to initiate early discussions related to expectations around timing of pregnancy and implications for treatment. It is critical to include individualized risk assessment when having these discussions as our data suggest that understanding potential risk of recurrence in concrete terms may impact and modulate one’s desire for future pregnancy, timing of pregnancy, and preference for surrogate vs. own pregnancy which can require years of planning and saving. Even though interest in having future children was not associated with standard prognostic risk factors, it was influenced by one’s understanding of their individual risk of recurrence when presented with hypothetical scenarios. Nevertheless, 17% of patients remained committed to having a biologic child even when presented with the hypothetical situation of near certain recurrence. It is important for providers to enter these conversations openly, understanding that when it comes to decisions regarding having a child after a breast cancer diagnosis, the balance of risk and benefit is highly personal and individual.

## Data Availability

The datasets generated during and/or analyzed during the current study are not publicly available due to ongoing follow-up analyses but are available from the corresponding author on reasonable request.
